# Skeletal muscle IL‐6 and regulation of liver metabolism during high‐fat diet and exercise training

**DOI:** 10.14814/phy2.12788

**Published:** 2016-05-15

**Authors:** Jakob G. Knudsen, Ella Joensen, Lærke Bertholdt, Henrik Jessen, Line van Hauen, Juan Hidalgo, Henriette Pilegaard

**Affiliations:** ^1^ Centre for Inflammation and MetabolismDepartment of BiologyUniversity of CopenhagenCopenhagenDenmark; ^2^ Universidad de Autonoma de BarcelonaCatalunyaSpain

**Keywords:** Gluconeogenesis, glucose oxidation, lipid oxidation, lipogenesis

## Abstract

Interleukin (IL)‐6 is released from skeletal muscle (SkM) during exercise and has been shown to affect hepatic metabolism. It is, however, unknown whether SkM IL‐6 is involved in the regulation of exercise training‐induced counteraction of changes in carbohydrate and lipid metabolism in the liver in response to high‐fat diet (HFD) feeding. Male SkM‐specific IL‐6 KO (MKO) and Floxed mice were subjected to Chow diet, HFD or HFD combined with exercise training (HFD ExTr) for 16 weeks. Hepatic phosphoenolpyruvate carboxykinase (PEPCK) protein content decreased with both HFD and HFD ExTr in Floxed mice, but increased in IL‐6 MKO mice on HFD. In addition, the intrahepatic glucose concentration was in IL‐6 MKO mice higher in HFD than chow. Within HFD ExTr mice, hepatic glucose‐6‐phosphatase (G6Pase) 36 kDa protein content was higher in IL‐6 MKO than Floxed mice. Hepatic pyruvate dehydrogenase kinase (PDK) 4 and PDK2 protein content was in Floxed mice lower in HFD ExTr than Chow. In addition, hepatic ACC1‐phosphorylation was higher and ACC1 protein lower in HFD. Together this suggests that SkM IL‐6 regulates hepatic glucose metabolism, but does not seem to be of major importance for the regulation of oxidative capacity or lipogenesis in liver during HFD or HFD combined with exercise training.

## Introduction

It has been shown that hepatic triglyceride (TG) content is increased when rodents are fed a high‐fat diet (HFD) (Rector et al. 2008; Gollisch et al. 2009) and is higher in obese than lean human subjects (Fabbrini et al. 2010). Such accumulation of intracellular lipids has been suggested to induce hepatic insulin resistance and reduce hepatic oxidative capacity (Gollisch et al. 2009), potentially leading to metabolically related diseases.

Physical activity has been shown to prevent HFD‐induced development of metabolic diseases (Rector et al. 2008; Gollisch et al. 2009). Although regulation of skeletal muscle (SkM) metabolism contributes to the exercise training‐induced counteraction of HFD feeding, the liver has also been reported to respond to exercise training (Rector et al. 2008; Gollisch et al. 2009), with potential effects on whole body metabolism. Hence, it has been shown that liver phosphoenolpyruvate carboxy kinase (PEPCK), cytochrome c, and cytochrome c oxidase subunit 1 (COX1) protein content increased with exercise training in mice on a chow diet (Haase et al. 2011). In addition, hepatic acetyl‐CoA carboxylase (ACC) protein content has been reported to decrease after exercise training in rats with fatty liver (Rector et al. 2008). Taken together these findings suggest that fatty acid (FA) uptake into mitochondria as well as oxidative and gluconeogenic capacity in the liver is increased with exercise training indicating that exercise training may rectify the hepatic metabolic imbalance caused by HFD. However, the potential endocrine mechanisms underlying the exercise training‐induced improvements in hepatic metabolism are unclear.

The plasma concentration of interleukin (IL)‐6 increases during exercise (Ostrowski et al. 1998; Steensberg et al. 2000; Pedersen et al. 2011) and IL‐6 has been suggested to exert effects in various tissues including the liver (Adser et al. 2011; Pedersen et al. 2011; Brandt et al. 2012; Knudsen et al. 2014, 2015). Thus, infusion of IL‐6 in humans during low‐intensity exercise has been shown to increase glucose rate of appearance (Febbraio et al. 2004). In accordance, a single injection of IL‐6 in rats has been reported to increase hepatic PEPCK mRNA content (Banzet et al. 2009), whereas another study demonstrated that exercise‐induced mRNA responses of gluconeogenic enzymes was maintained in IL‐6 knockout (KO) mice (Fritsche et al. 2010). In addition, a study has shown that IL‐6 KO mice on HFD have reduced hepatic 3‐hydroxyacyl‐CoA dehydrogenase (HAD) activity, reduced carnitine palmitoyl transferase 1 (CPT1) mRNA and reduced protein content of the respiratory chain complexes compared with wild‐type (WT) mice on HFD (Matthews et al. 2010). Taken together this indicates that IL‐6 is central in the regulation of lipid metabolism and oxidative capacity in liver and suggests that IL‐6 may be required for exercise training‐induced adaptations in liver metabolism. However, the role of SkM IL‐6 in the regulation of liver metabolism in response to exercise training remains unknown. Thus, this study was performed to test the hypothesis that SkM IL‐6 is required for the exercise training‐induced counteraction of HFD‐associated changes in carbohydrate metabolisms and lipid storage in the liver.

## Methods

### Animals

The mice used in this experiment were fully backcrossed C57BL6 mice carrying either a LoxP insert flanking the second exon of the IL‐6 gene (Floxed) as previously described (Quintana et al. 2013) or IL‐6 MKO mice carrying Cre‐recombinase under the control of the myogenin promoter (Li et al. 2005) in addition to the LoxP insert. SkM‐specific knockout of IL‐6 was confirmed on genomic DNA from SkM and liver with primers surrounding the floxed region of the IL‐6 gene (Knudsen et al. 2015). Basal plasma IL‐6 levels showed high variability and were rarely detectable in both genotypes, and no differences were observed between Floxed and IL‐6 MKO mice (data not shown). The SkM IL‐6 mRNA content has previously been shown to be reduced in the IL‐6 MKO relative to Floxed mice (Ferrer et al. 2014; Knudsen et al. 2015). The mice were housed at 22°C and a 12:12 hour light: dark cycle with ad libitum access to standard rodent chow and water, until the onset of the experiment. Fat mass, lean mass, and body weight as well as glucose and insulin tolerance and skeletal muscle IL‐6 mRNA content from these mice have previously been published (Knudsen et al. 2015).

### Ethics statement

The experiments were approved by and conducted in accordance with guidelines provided by The Danish Animal Experiments Inspectorate (The Danish Ministry of Foods, Agriculture and Fisheries, Denmark).

### Experimental protocol

Male Floxed and IL‐6 MKO mice were at the age of 8 weeks divided in three groups. A group receiving standard rodent chow (Altromin 1314F, Brogaarden, Lynge, Denmark) (Chow), a group receiving a high‐fat diet (HFD) with 60% of calories from fat (Altromin C1090‐60, Brogaarden, Lynge, Denmark), and a group receiving HFD in combination with having access to a running wheel (diameter 24.2 cm, Mini‐mitter) (HFD ExTr) for 16 weeks (*n* = 10). The running wheels were blocked occasionally ensuring that the two exercise training groups were running a similar distance (~ 20 km/week, with an average pace of 26.0 ± 1.6 m/min for Floxed and 23.7 ± 0.4 m/min for IL‐6 MKO). Additionally, to increase the exercise load and increase adaptation to exercise, HFD ExTr mice were during the last 4 weeks running on a treadmill (303401 series, TSE systems, Germany) 60 min 3 days a week at 13.8 m/min and 10° incline the first week increasing to 16.2 m/min and 10° incline the last week. At the end of the experimental period, the running wheels were blocked 24 h before euthanization. Ad libitum access to food and water was maintained. The mice were euthanized by cervical dislocation and livers were removed and snap frozen in liquid nitrogen. Trunk blood was collected and centrifuged at 2600 *g*, 4°C for 15 min to generate plasma. All samples were then stored at −80°C.

### Plasma metabolites

Plasma glucose and plasma lactate were measured by enzymatic assays as previously described (Li et al. 2005) and plasma *β*‐hydroxybutyrate was measured using an enzymatic kit according to manufacturer's protocol (# MAK041, Sigma aldrich, Copenhagen, Denmark).

### Hepatic glucose, lactate, glycogen, and triglycerides

Liver samples were crushed in liquid nitrogen and liver glucose and lactate content was determined fluorometrically (Lowry and Passonneau 1972). To determine hepatic glycogen content, liver samples were hydrolyzed by boiling for 2 h in 1 mol/l HCL and glycosyl units were determined fluorometrically as previously described (Lowry and Passonneau 1972). Liver triglyceride (TG) content was measured as previously described (Norris et al. 2003). Briefly, 10 mg tissue was treated with KOH:ethanol overnight, cell debris was removed by centrifugation, and glycerol was measured using Free Glycerol Reagent (Sigma Aldrich, Denmark) according to the manufacturers protocol.

### Hepatic β‐HAD activity

Hepatic L‐3‐hydroxyacyl‐CoA dehydrogenase (*β*‐HAD) activity was measured as previously described (Essengustavsson and Henriksson 1984). Briefly 10 mg of crushed liver tissue was homogenized in 0.3 mmol/l phosphate buffer containing 0.05% BSA. *β*‐HAD activity was calculated from the conversion of NADH to NAD in the presence of 5.87 mmol/l S‐Acetoacetyl‐CoA and adjusted to the protein concentration determined using the bicinchoninic acid (BCA) assay.

### RNA isolation and Reverse transcription

RNA was isolated as previously described (Chomczynski and Sacchi 1987) with modifications (Pilegaard et al. 2000) and cDNA was produced by reverse transcribing 3 *μ*g of RNA using superscript II reverse transcriptase (Invitrogen, Thermofisher Scientific, Naerum, Denmark) as previously described (Pilegaard et al. 2000).

### Real time PCR

Content of specific mRNAs was determined using real‐time PCR (ABI 7900 Prism, Applied Biosystems, Foster City, MA). Primers and Taqman Probes recognizing mouse PGC‐1*α* (FP: 5′ CTCCCTTGTATGTGAGATCACGTT 3′; RP 5′ TGCGGTATTCATCCCTCTTGA 3′; Taqman Probe: 5′ ACAGCCGTAGGCCCAGGTACGACA 3′), were designed from mouse cDNA sequences (Entrez‐NIH and Ensembl, Sanger Institute) using primer express (Applied Biosystems, Paisley, UK) and obtained from TAG Copenhagen (Copenhagen, Denmark). The probe was 5′ 6‐carboxyflouresine (FAM) and 3′ 6‐carboxy‐N, N, N’, N’‐ tetramethylrhodamine (TAMRA) labeled. Predeveloped primers and Taqman probe were used to detect GAPDH (Applied Biosystems). A serial dilution was created from a pool of all samples and used to construct a standard curve to calculate the mRNA content of PGC‐1*α* and GAPDH in the individual samples. PGC‐1*α* mRNA was normalized to GAPDH mRNA content, which was not affected by the intervention or genotype.

### Lysate generation and western blotting

Lysates were generated from ~20 mg of crushed liver as previously described (Birk and Wojtaszewski 2006) except that homogenization was performed in a Tissue Lyser (Qiagen, Hilden, Germany) for 2 min at 30 Hz. Homogenized samples were left for 1 h at 4°C turning end over end and lysates were generated by centrifugation at 16000 *g*, 4°C for 20 min. Protein concentrations of lysates were determined using the BCA assay (Thermofisher Scientific) and samples were adjusted to 2 μg/μl in sample buffer containing SDS. Proteins were separated by SDS PAGE and transferred to a PVDF membrane by semidry blotting. The membrane was blocked in 3% fish gel (FG) and probed over night with primary antibodies recognizing ACC1^Ser79^ and 2^Ser212^ phosphorylation (#07‐303, Millipore, Darmstadt, Germany), ACC1 and 2 (Streptavidin‐HRP, Dako, Denmark), respiratory chain complexes I‐V (#110413, ABCAM, Cambridge, UK), PEPCK (#10004943, Cayman chemicals, Ann Abor, MI), PDK2 (# ST1643, Calbiochem, Millipore), PDK4 (kind gift from professor Grahame Hardie, University of Dundee, UK), G6Pase (#27198, Santa Cruz biotechnology, Dallas, TX) and GAPDH (# 2118, Cell Signaling Technologies, Beverly, MA). The membrane was the following day incubated in horseradish peroxidase conjugated secondary antibody in 3% FG recognizing the primary antibody. Bands were visualized using chemiluminescence (Luminata class1co/forte ECl reagent, Millipore, Damstad, Germany) and the Image Quant LAS 4000 system (GE Healthcare, Hatfield, UK) and quantified using Image Quant TL (GE Healthcare).

### Statistics

All values are presented as mean ± standard error of the mean (SE). Two way analysis of variance (ANOVA) was used to test effects of intervention and genotype. In addition, a one‐way ANOVA was used to determine the effect of interventions within each genotype separately. A student Newman–Keuls test was used to locate differences between groups. If variances were not equal in the two way ANOVA, a Student's *t*‐test was used to determine effects of genotype and if variances were not equal in the one way ANOVA, an ANOVA on RANKS was used to test the effect of the intervention. A value of *P* < 0.05 was considered significant.

## Results

### Basal plasma parameters

To investigate if SkM IL‐6 affected whole body glucose metabolism, plasma glucose, lactate, and *β*‐hydroxybutyrate levels were determined. Neither HFD, HFD, and exercise training nor loss of SkM IL‐6 affected plasma glucose, lactate or *β*‐hydroxybutyrate levels (Table 1).

**Table 1:** Plasma concentration of glucose, lactate, and β‐hydroxybutyrate in Floxed and IL‐6 MKO mice after 16 weeks on Chow, HFD, or HFD with exercise training (*n* = 9–10). Values are mean ± SE

	Chow		HFD		HFD ExTr	
	Floxed	IL‐6 MKO	Floxed	IL‐6 MKO	Floxed	IL‐6 MKO
Plasma glucose (mmol/l)	5.1 ± 0.2	5.0 ± 0.2	5.1 ± 0.2	5.1 ± 0.2	4.9 ± 0.2	4.8 ± 0.2
Plasma lactate (mmol/l)	4.3 ± 0.3	4.3 ± 0.2	4.7 ± 0.4	3.9 ± 0.3	4.2 ± 0.3	4.3 ± 0.2
Plasma *β*‐hydroxybutyrate (mmol/l)	1.3 ± 0.1	1.4 ± 0.2	1.5 ± 0.1	1.5 ± 0.1	1.4 ± 0.1	1.4 ± 0.1

### Hepatic glucose, lactate, glycogen, and triglycerides

**Figure 1:**
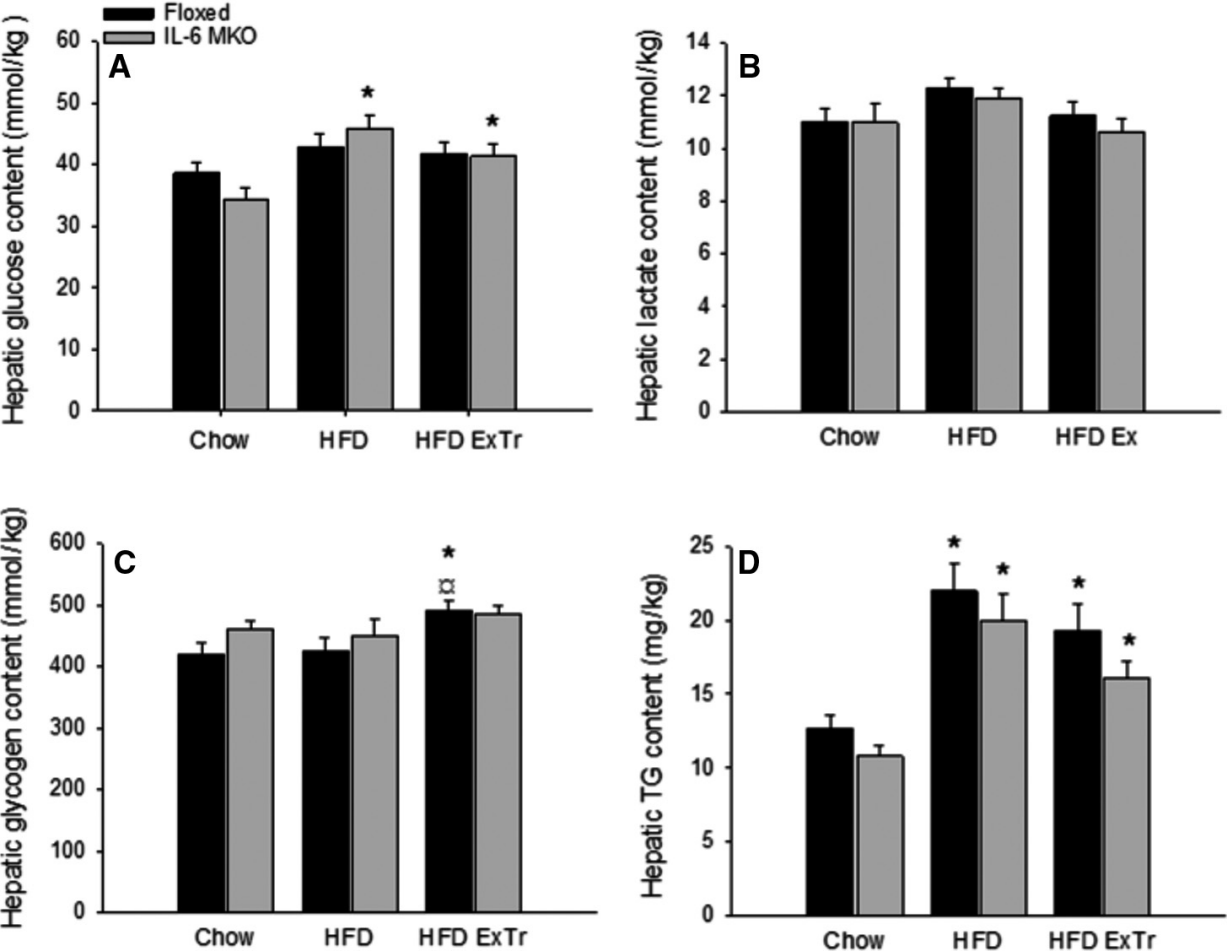
Hepatic glucose (A), lactate (B), glycogen (C), and triglyceride (TG) (D) content in Floxed and IL‐6 MKO mice after 16 weeks on Chow, HFD or HFD with exercise training (*n* = 9–10). Values are mean ± SE.*Significantly different from Chow within given genotype (*P* < 0.05); ^¤^Significantly different from HFD within given genotype (*P* < 0.05).

To investigate if SkM IL‐6 affected liver carbohydrate metabolism, liver glucose, lactate, glycogen, and triglycerides were investigated. While hepatic glucose concentration was ~30% higher (*P* < 0.05) in HFD and HFD ExTr than Chow within IL‐6 MKO mice (Fig. 1A), the lactate concentration did not change with HFD or HFD combined with exercise training in either genotype (Fig. 1B). In addition, Floxed mice had ~10% higher (*P* < 0.05) hepatic glycogen content in HFD ExTr than Chow and HFD (Fig. 1C). Hepatic TG content was ~2 fold and ~1.5 fold higher (*P* < 0.05) in HFD and HFD ExTr, respectively, than Chow for both genotypes (Fig. 1D).

### Hepatic gluconeogenic capacity

To investigate the importance of SkM IL‐6 in exercise training‐induced counteraction of changes in key factors in gluconeogenesis with HFD, the protein content of gluconeogenic enzymes and regulators of substrate choice was measured.

**Figure 2:**
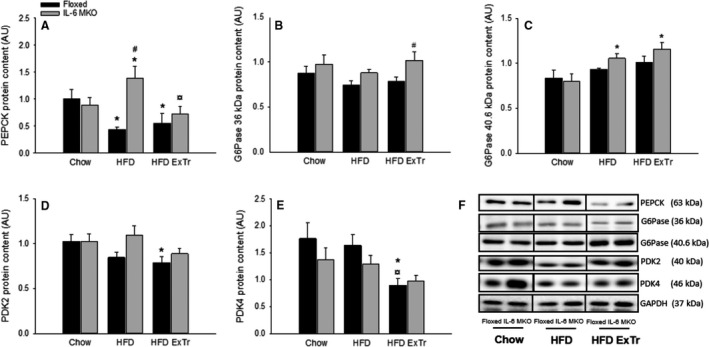
Hepatic PEPCK (A), G6Pase 36 kDa (B), G6Pase 40.5 kDa (C), PDK2 (D) PDK4 (E) protein content and representative blots (F) in Floxed and IL‐6 MKO mice after 16 weeks on Chow, HFD or HFD with exercise training (*n* = 9–10). Values are mean ± SE. *Significantly different from Chow within given genotype (*P* < 0.05); ^¤^Significantly different from HFD within given genotype (*P* < 0.05); ^#^Significantly different from Floxed mice within given intervention (*P* < 0.05).

Liver PEPCK protein content was in Floxed mice ~50% lower (*P* < 0.05) in HFD and HFD ExTr than Chow and in IL‐6 MKO ~1.2 fold higher (*P* < 0.05) in HFD than Chow and ~50% lower (*P* < 0.05) in HFD ExTr than HFD (Fig. 2A). Thus, hepatic PEPCK protein content was ~2 fold higher (*P* < 0.05) in IL‐6 MKO than Floxed mice within the HFD mice.

The content of the G6Pase 36 kDa subunit was unchanged in HFD and HFD ExTr compared with Chow in both genotypes. However, liver G6Pase 36 kDa protein content was ~1.2 fold higher (*P* < 0.05) in IL‐6 MKO than Floxed mice within HFD ExTr (Fig. 2B). The liver protein content of G6Pase 40.5 kDa was higher (*P* < 0.05) in HFD and HFD ExTr than Chow within IL‐6 MKO mice (Fig. 2C), however no significant differences were present in PDK2 and PDK4 between groups within IL‐6 MKO mice.

Within Floxed mice, hepatic PDK2 protein content was ~20% lower (*P* < 0.05) in HFD ExTr than Chow (Fig. 2D) and hepatic PDK4 protein content was ~50% lower (*P* < 0.05) in HFD ExTr than both HFD and Chow (Fig. 2E).

### Hepatic fatty acid metabolism

Phosphorylation and protein content of key factors in lipogenesis and mitochondrial fatty acid uptake were determined to examine the effect of SkM IL‐6 on fat metabolism in liver.

**Figure 3:**
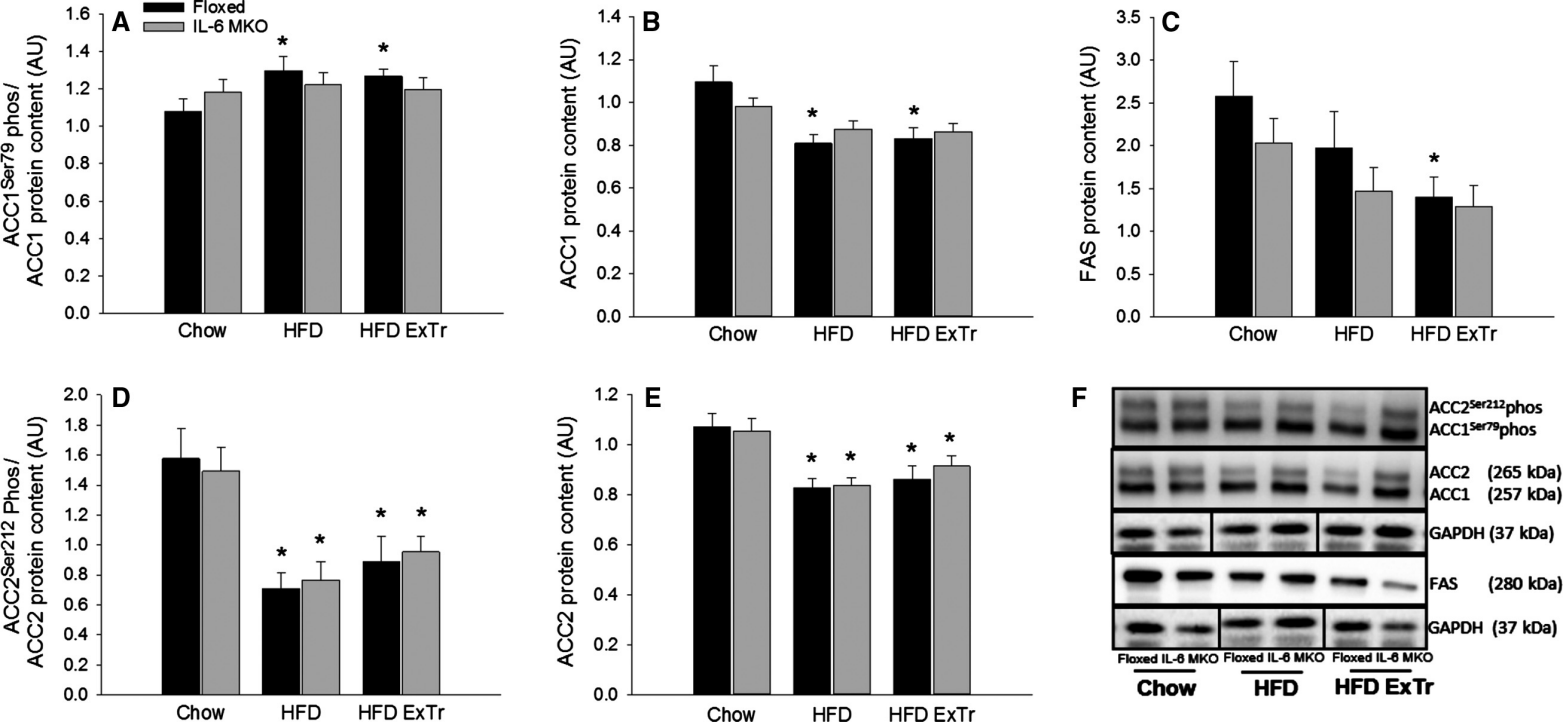
Hepatic ACC1^Ser79^ phosphorylation normalized to ACC1 protein content (A), ACC1 protein content (B), FAS protein content (C), ACC2^Ser212^ phosphorylation normalized to ACC2 protein content (D), ACC2 protein content (E) and representative blots (F) in Floxed and IL‐6 MKO mice after 16 weeks on Chow, HFD or HFD with exercise training (*n* = 9–10). Values are mean ± SE. *Significantly different from Chow within given genotype (*P* < 0.05).

ACC1^Ser79^ phosphorylation in liver was unaltered in both Floxed and IL‐6 MKO mice, whereas ACC1 protein content was ~20% lower (*P* < 0.05) in HFD and HFD ExTr than Chow within Floxed mice. This resulted in ~20% higher (*P* < 0.05) ACC1^Ser79^ phosphorylation normalized to ACC1 protein content in HFD and HFD ExTr than Chow in Floxed mice (Fig. 3A and B) with no effects in IL‐6 MKO mice.

Fatty acid synthase (FAS) protein content in the liver was ~40% lower (*P* < 0.05) in HFD ExTr than Chow within Floxed mice (Fig. 3C) with no effects in IL‐6 MKO mice.

Hepatic ACC2^Ser212^ phosphorylation was ~50–70% lower (*P* < 0.05) and ACC2 protein content was ~20% lower (*P* < 0.05) in HFD and HFD ExTr than Chow in both genotypes. This caused ACC2^Ser212^ phosphorylation normalized to ACC2 protein content to be ~40–50% lower (*P* < 0.05) in HFD and HFD ExTr than Chow (Fig. 3D and E).

Neither HFD, HFD and exercise training nor loss of SkM IL‐6 affected the hepatic *β*‐HAD activity (Table 2).

**Table 2:** Hepatic HAD activity in Floxed and IL‐6 MKO mice after 16 weeks on Chow, HFD, or HFD with exercise training (*n* = 5–8). Values are mean ± SE

	Chow		HFD		HFD ExTr	
	Floxed	IL‐6 MKO	Floxed	IL‐6 MKO	Floxed	IL‐6 MKO
HAD activity (mmol/min/mg protein)	5.5 ± 0.8	6.3 ± 0.8	5.6 ± 1.0	4.5 ± 0.9	4.7 ± 1.0	4.3 ± 0.8

### Hepatic mitochondrial capacity

The protein content of respiratory chain complexes and PGC‐1*α* mRNA content were determined to investigate the effect of SkM IL‐6 on the oxidative capacity of the liver.

**Figure 4:**
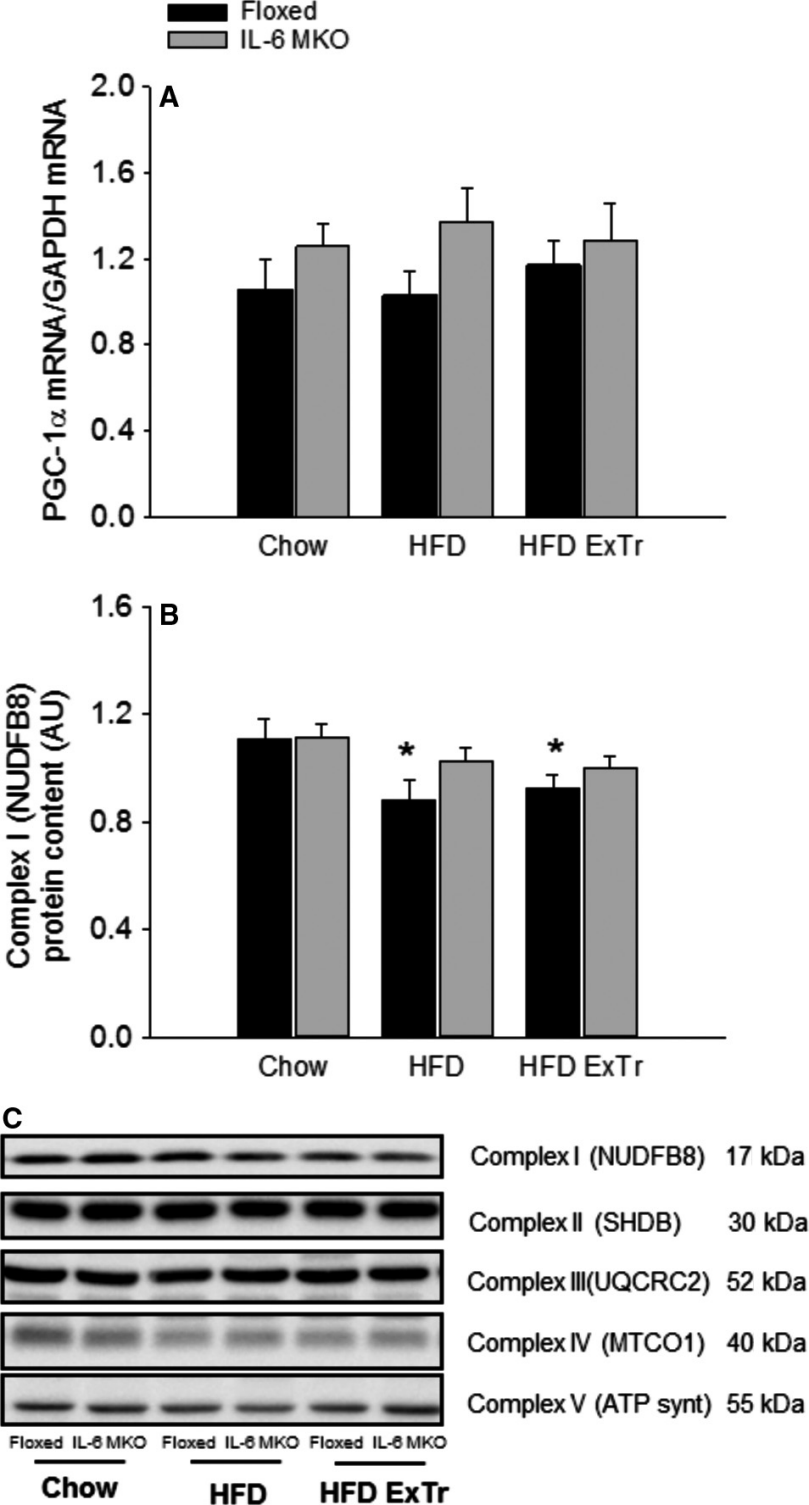
Hepatic PGC‐1*α* mRNA content (A) and respiratory chain complex protein content (B,C) in Floxed and IL‐6 MKO mice after 16 weeks on Chow, HFD or HFD with exercise training (*n* = 9–10). Values are mean ± SE. *Significantly different from Chow within given genotype (*P* < 0.05).

Hepatic PGC‐1*α* mRNA content was unaltered in response to HFD and HFD combined with exercise training in both genotypes (Fig. 4A).

The protein content of complex I of the respiratory chain was in the liver of Floxed mice ~20% lower (*P* < 0.05) in HFD and HFD ExTr than Chow, but otherwise no differences were observed in any of the other respiratory chain complexes either in Floxed or IL‐6 MKO mice (Fig. 4B, C).

## Discussion

The main finding of this study is that SkM IL‐6 seems to contribute to the regulation of markers of gluconeogenesis in response to HFD, without playing a major role in the regulation of hepatic fat metabolism or oxidative capacity in mice on HFD or HFD combined with exercise training.

As previously reported (Knudsen et al. 2015) the increased fat mass and reduced glucose tolerance in the mice from this study demonstrate that the 16 weeks of HFD were effective although total body weight and insulin tolerance were unaffected. In addition, the previously published observation (Knudsen et al. 2015) that both genotypes reduced fat mass when HFD was combined with exercise suggests that the exercise training protocol did counteract some effects of the HFD. The present finding that ACC2 phosphorylation was decreased in Floxed mice with 16 weeks of HFD feeding, without changes in insulin tolerance (Knudsen et al. 2015), may suggest that the liver was using glucose rather than FA as substrate for oxidation when the mice were on HFD. Furthermore, the increased ACC1 phosphorylation and reduced PEPCK protein content in the Floxed mice on HFD may suggest that de novo lipogenesis and gluconeogenic capacity were reduced with HFD feeding. Together these findings strongly suggest that the 16 weeks of HFD changed the regulation of hepatic metabolism. Furthermore, the exercise training‐induced increase in hepatic glycogen content and decrease in PDK4 and PDK2 protein content in Floxed mice provide evidence for exercise training‐induced modifications of hepatic carbohydrate metabolism when on a HFD. These results also indicate that HFD combined with exercise training caused the liver to become more dependent on glucose as substrate than HFD alone. Additionally, the observation that exercise training reduced hepatic FAS protein content in Floxed mice on HFD further supports that the exercise training protocol used in this study induced changes in hepatic metabolism.

The hypothesis of this study was that SkM IL‐6 plays a role in the exercise training‐induced counteraction of changes in liver carbohydrate and lipid metabolism caused by HFD. The present observations that glucose accumulated in the liver of IL‐6 MKO mice in response to HFD may suggest an impairment of intrahepatic glucose handling when SkM IL‐6 was lacking. Accordingly, PEPCK protein content was increased in response to HFD in IL‐6 MKO mice suggesting that the increased intrahepatic glucose content in IL‐6 MKO mice may be a result of an increased gluconeogenic activity. The increased level of PEPCK in IL‐6 MKO mice on HFD indicates that SkM IL‐6 is required for the reduction in hepatic PEPCK protein content observed with HFD. This is in contrast to the previous findings that injection of IL‐6 leads to increased PEPCK mRNA (Banzet et al. 2009) but this observation was made in chow fed rats and it could be suggested that either species or diet may influence the effect of IL‐6. The observations that G6Pase 36 kDa protein content increased in IL‐6 MKO mice compared with Floxed mice and G6Pase 40.5 kDa increased only in IL‐6 MKO mice with HFD and HFD ExTr support that gluconeogenic capacity may have been higher in IL‐6 MKO than Floxed mice when on HFD and HFD ExTr. At the same time, the similar PDK2 and 4 protein content in IL‐6 MKO and Floxed mice suggests that skeletal muscle IL‐6 did not influence basal hepatic carbohydrate oxidation. As the plasma glucose and hepatic glycogen content was similar in chow, HFD and HFD ExTr within IL‐6 MKO mice, it may be speculated that an impairment in glucose transport out of the liver contributed together with an enhanced gluconeogenesis lead to the increased hepatic glucose concentration in IL‐6 MKO mice on HFD.

It has previously been shown that whole body IL‐6 KO mice have a reduced capacity for hepatic *β*‐oxidation when on HFD (Matthews et al. 2010). The present finding that both Floxed and IL‐6 MKO mice had reduced ACC2^Ser212^ phosphorylation when on HFD suggests that there was a higher potential for uptake of FA in to mitochondria in the liver independent of SkM IL‐6.

The observed increase in ACC1^Ser79^ phosphorylation in response to HFD and HFD combined with exercise only in Floxed mice may on the other hand raise the possibility that SkM IL‐6 alters ACC1 activity. As the major role of ACC1 is considered to be production of malonyl‐CoA for FA synthesis (Abu‐Elheiga et al. 2005), it may be speculated that this reflects that lipogenesis was increased with HFD and HFD ExTr in an IL‐6‐dependent manner. However, as ACC1 protein content was decreased in floxed mice on HFD and HFD combined with exercise training, the amount of active ACC1 protein would be lower in mice on HFD and HFD ExTr than on chow, and thus indicate that lipogenesis was in fact decreased rather than increased in a skeletal muscle IL‐6‐dependent manner. This is also supported by the reduction in FAS protein content in HFD ExTr in Floxed mice. However, the similar increase in hepatic TG content with HFD and HFD ExTr in the two genotypes, does not provide evidence that SkM IL‐6 plays a major role in the regulation of liver TG accumulation in response to HFD with or without exercise training.

The present findings that SkM IL‐6 deficiency had no effect on oxidative capacity in the liver do not support previous observations that oxidative capacity was reduced with HFD in whole body IL‐6 Knockout mice (Matthews et al. 2010). Rather, IL‐6 MKO mice seemed to be protected from the mild reduction in complex I observed in Floxed mice in response to HFD. However, it may be speculated that redundancy via IL‐6 released from other tissues could explain the discrepancies between the present and previous findings (Matthews et al. 2010).

In conclusion, these results suggest that SkM IL‐6 is contributing to decreased hepatic glucose production in response to HFD through regulation of hepatic PEPCK and G6Pase protein content. However, SkM IL‐6 does not seem to be of major importance for oxidative capacity, lipogenesis or the regulation of mitochondrial fatty acid uptake in liver during HFD or HFD combined with exercise training

## Conflict of Interest

The authors have nothing to disclose
